# Complete Genome Sequence of Vibrio cholerae O1 El Tor Strain C6706

**DOI:** 10.1128/MRA.01301-20

**Published:** 2021-01-21

**Authors:** Yuding Weng, X. Renee Bina, James E. Bina

**Affiliations:** a University of Pittsburgh School of Medicine, Department of Microbiology and Molecular Genetics, Pittsburgh, Pennsylvania, USA; Loyola University Chicago

## Abstract

Vibrio cholerae is a global health threat and a model enteric pathogen that causes the human disease cholera. Here, we report the complete genome sequence of the seventh-pandemic V. cholerae O1 El Tor strain C6706.

## Abstract (precis)

Vibrio cholerae is a global health threat and a model enteric pathogen that causes the human disease cholera. Here, we report the complete genome sequence of the seventh pandemic V. cholerae O1 El Tor strain C6706.

## ANNOUNCEMENT

Vibrio cholerae is an aquatic organism and facultative human pathogen that causes the epidemic disease cholera. Cholera is an acute diarrheal disease that has been affecting humans since antiquity (reviewed in reference 1). There have been seven cholera pandemics since the 1800s, with the seventh and ongoing pandemic beginning in 1961. The pandemic disease is caused by toxigenic V. cholerae strains belonging to the O1 serogroup harboring horizontally acquired genes encoding two critical virulence factors, the toxin-coregulated pilus and cholera toxin (2). Toxigenic V. cholerae strains are separated into two biotypes, the classical biotype and the El Tor biotype. Classical strains were responsible for the first six cholera pandemics. The seventh and ongoing pandemic was caused by the El Tor biotype, which emerged in 1961 and has now displaced the classical biotype as the main cause of pandemic cholera. Here, we present the complete genome sequence of the seventh-pandemic V. cholerae O1 El Tor strain C6706, which was isolated from a patient in Peru in 1961. Strain C6706 is widely used to study V. cholerae biology, and its genome sequence will facilitate future studies of V. cholerae evolution and pathogenesis.

V. cholerae strain C6706, originally isolated by the Centers for Disease Control and Prevention (3), was generously provided by Ankur Dalia, Department of Biology, Indiana University, and confirmed to be naturally competent (4). This strain has been maintained at −80°C in 20% glycerol since its receipt. To sequence the V. cholerae C6706 genome, total genomic DNA was isolated from a saturated lysogeny broth culture using the MasterPure kit (Epicentre, Madison, WI) according to the manufacturer’s instructions. The total genomic DNA was then sent to SNPsaurus (Eugene, OR) for whole-genome PacBio sequencing. To accomplish this, genomic DNA was processed according to the PacBio “Preparing Multiplexed Microbial Sequel Single Molecule Real-Time (SMRT) Bell Libraries for the PacBio Sequel System” protocol and sequenced with SMRT Link 6.0 sequencing chemistry and a library size selected for fragments of ≥7 kb with a 30-hour read time using a fraction of a SMRT cell. DNA size selection was performed on the genomic DNA without additional shearing with a BluePippin system (Sage Sciences), and the sequencing library was prepared using a PacBio SMRTbell Express template prep kit 2.0. This resulted in 285,997 sequencing reads with a 7,645-bp mean read length to generate 2.2 × 109 total bases that were *de novo* assembled and polished using Flye 2.8 (5) with the default settings. The total assembled length was 4,085,397 bp, with a read depth for the assembled genome of 506×. Flye 2.8 identified two closed circular contigs of 3,015,051 bp and 1,070,346 bp. The assembly quality was assessed using BUSCO 3 and was 99.2% complete, with 98.4% of the genome single copy and 0.8% duplicated (6).

The 3.01- and 1.07-Mb contigs, representing the V. cholerae large and small chromosomes (7), had a GC content of 47.6% and 46.9%, respectively. The genome was annotated using the NCBI Prokaryotic Genome Annotation Pipeline 4.13 (8), which revealed wild-type alleles for the *hapR* and *luxO* quorum sensing-related genes as previously described (4).

### Data availability.

The C6706 genome sequence was deposited in GenBank under BioProject PRJNA675358 with the following accession numbers: CP064350 and CP064351. The raw sequencing results were deposited into the NCBI SRA database under the accession number SRR13006676.
